# Using Narrative Inquiry to Understand Anti-Muslim Racism in Canadian Nursing

**DOI:** 10.1177/08445621221129689

**Published:** 2022-09-29

**Authors:** Nasrin Saleh, Nancy Clark, Anne Bruce, Mehmoona Moosa-Mitha

**Affiliations:** 1 School of Nursing, 8205 University of Victoria, Victoria, BC, Canada; 2 School of Social Work, 8205 University of Victoria, Victoria, BC, Canada

**Keywords:** Islamophobia, anti-muslim racism, hijab, narrative inquiry, composite narratives, intersectionality

## Abstract

**Background:**

Islamophobia or, anti-Muslim racism, and more specifically, gendered islamophobia targeting Muslim women who wear a hijab is rising globally and is aggravated by the COVID-19 pandemic. However, anti-Muslim racism is not well understood in Canadian nursing.

**Purpose:**

This study utilized narrative inquiry to understand anti-Muslim racism through the experiences of nurses who wear a hijab with the goal of putting forward their counter-narrative that disrupts anti-Muslim racism in Canadian nursing.

**Methods:**

Narrative inquiry informed by Critical Race Feminism, care ethics, and intersectionality were used to analyze the factors shaping anti-Muslim racism and composite narratives were used to present the results.

**Results:**

The three composite narratives are: ‘This is Who I Am: A Muslim Nurse with a Hijab and an Accent’; ‘I Know What is at Play: Unveiling Operating Power Structures and Power Relations’; and ‘Rewriting the Narrative: Navigating Power Structures and Power Relations’. These composite narratives constituted the nurses’ counter-narrative. They revealed intersections of gendered, racial divisions of labour and religious narratives that shape anti-Muslim racism, as operating power relations in nursing, and how Muslim nurses reclaimed control to resist their racialized stereotypes.

**Conclusion:**

Findings suggest that anti-Muslim racism in nursing operates through multiple intersecting power relations. Using stories can mobilize transformational change so that anti-racist practices, policies, and pedagogy can be embraced.

## Background and purpose

Islam is one of the fastest growing religions in Canada and is the second largest after Christianity, representing 3.7% of the population (Statistics Canada, 2021). Yet, Canadian Muslims are often stereotyped as immigrants who do not fit within the fabric of Canada (Environics Institute, 2016). Such erroneous narratives create ongoing challenges for Canadian Muslims as a result of oversimplified stereotypes and assumptions that infiltrate public opinion and institutions and which are enforced by policies and laws targeting Muslims (Environics Institute, 2016; McDonough, 2003). Islamophobia is thus a daily reality for Canadian Muslims and is a particular concern to Muslim women who wear a hijab due to their hypervisibility (Litchmore & Safdar, 2016; Rahmath et al., 2016).

Islamophobia is the presumption that Muslims are “inherently violent, alien, and inassimilable” (Beydoun, 2016, p. 2), and is characterized by a dialectic process in which exclusionary state policies perpetuate such presumptions and emboldens animus towards Muslims. During the COVID-19 pandemic, islamophobia globally rose due to messages and posts on social media platforms that connected Muslim communities with increased rates of infection and characterized Muslims as super spreaders of the virus (Al-Qazzaz, 2020; Chandra et al., 2021). Although these online social media messages and posts were not directly connected to stereotypes of Muslims as violent or associated with terrorism, they put Muslims and their communities at risk of harm (Chandra et al., 2021). According to Riess and Lemos (2021), the killing of four members of a Muslim family in London, Ontario on June 6, 2021 is recent evidence of heightened Anti-Muslim racism. In this paper, we used Islamophobia and anti-Muslim racism interchangeably as both essentialize a group of people as having qualities that cast them as inferior (Moosavi, 2015).

Gendered Islamophobia refers to specific forms of racialized discrimination that affect Muslim women differently than Muslim men (Zine, 2006), which is reinforced by master-narratives about women who veil as agency-less, unfree strange, and dangerous (Thobani, 2007). For example, when the provincial legislature in Quebec banned public employees from wearing a hijab^1^ at work, the government relied on and reinforced the view that the hijab is a symbol of oppression that threatens Western values of freedom (Bilge, 2010; Khiabany & Williamson, 2008; Riley, 2011).

Societal stereotypes and social structures are not disconnected from everyday nursing practice. Some authors claim racial stereotypes operate in nursing under the guise that they could not exist alongside the discipline's core values of caring, empathy, and professionalism (Barbee, 1993; Hilario et al., 2018). Others suggest racism in nursing is fostered through White privilege and the construct of the ‘White good nurse’, which serves to privilege those who fit within such construct and marginalize those who do not, while claiming to upholding the discipline's moral commitments (Giddings, 2005; Louie-Poon et al., 2022). In addition to racism, sexism is a prevalent issue in nursing. Nursing is the most gendered discipline (about 90% of nurses are women), largely because caring and nurturing are attributes attached to women (DeVito, 2015; McLaughlin et al., 2009). Historically, nursing was seen as low paying work and nurses were considered physicians’ ‘handmaiden’ (Hoeve et al., 2014). Unfortunately, these are prevailing views (Gauci et al., 2022; Girvin et al., 2016).

There is minimal discussion of racism in nursing literature from the perspective of Muslim nurses. Little is known on how systemic processes of oppression, such as those in the context of COVID-19 or rooted in colonization, shape Canadian Muslim nurses’ experiences of racism. Further, gendered forms of anti-Muslim racism are even less likely to be studied and when they are, the focus is often on Muslim men or through a male gendered paradigm that renders Muslim women silent (Aziz, 2012; Molana, 2019; Strossen, 2007). Hence, we proposed that understanding the experiences of nurses who wear a hijab contributes to addressing this gap in nursing knowledge and offers a unique window into the interplay of multilevel power structures within nursing that sustain privilege and oppression. We also examined religion as a social marker, which is often neglected in intersectional studies (Reimer-Kirkham & Sharma, 2011). Finally, his study contributes to the nascent understanding about how anti-Muslim operates in nursing. Nurses’ stories are used to dismantle negative stereotypes about them and contribute to advancing social justice. Understanding how the hijab has been constructed within Canadian nursing adds to disrupting the public and political discourses that sustain Islamophobia and systemic subjugation (Tuyisenge & Goldenberg, 2021).

Therefore, the purpose of this study was to explore the experiences of Canadian nurses who wear a hijab, to share their stories of resistance, and to disrupt Islamophobia across everyday nursing practice, education, and policy.

## Methods and procedures

### Design

Qualitative research methods were used drawing on Riessman's (2008) approach to narrative inquiry. Riessman acknowledges the importance of contexts and the personal influence of the researcher on the analysis, therefore we conducted reflexive memos and discussions throughout the analysis. To represent the nurses’ counter-narratives, we constructed three composite narratives in first person. Presenting counter-narratives this way reflect actual life experiences and are composed as composites (Patton & Catching, 2009). Composite narratives are congruent with Riessman's (2008) approach of analyzing narratives holistically, as they present multiple situated accounts that are woven together (Willis, 2019).

This study was theoretically informed by Critical Race Feminism (CRF) and care ethics. We used Intersectionality as an analytical lens to underline how social identities, including religion, and social locations, matter in the broader context of racism in nursing. We discuss these theoretical approaches and how they offer insight into anti-Muslim racism. CRF is founded on the ontological assumption that race is socially constructed (Shih et al., 2007). It aims to challenge global feminism and address complexity by recognizing that the intersections of gender, race, and class uniquely impact the lives of women of color (Harris, 2003; Wing, 2003). As such, CRF examines how Whiteness shapes discourses, assumptions, and stereotypes that infiltrate institutional cultures, including nursing, and support unconscious biases and the exclusion of minorities (Delgado & Stefancic, 2013). A central tenet of CRF is the importance of counter-narratives that challenge normative views of race and gender to highlight the voices of women of color and dismantle racism (Harper et al., 2009; Montoya, 2006). We also used CRF as a sensitizing theory to explore how nurses who wear a hijab resist racial stereotypes and systemic racism and to question the gendered, racialized assumptions and power relations embedded in the nurses’ experiences.

**Figure 1.**
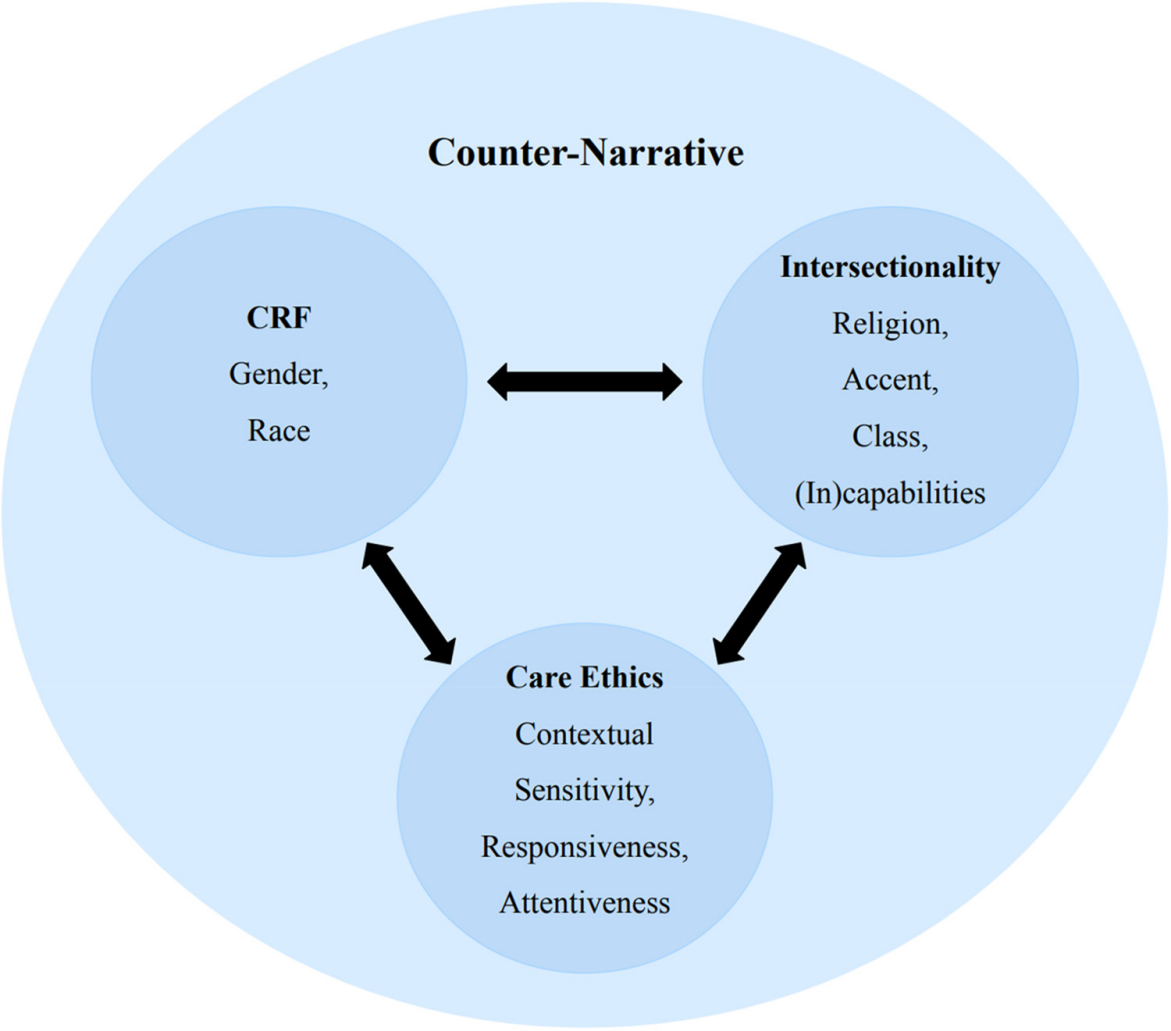
Application of critical theoretical approaches.

We used the political and moral discourse of feminist care ethics proposed by Hankivsky (2004), which foregrounds the concepts of contextual sensitivity, responsiveness, and attentiveness to the consequence of choice (Figure 1). From a care ethic perspective, people are shaped by their historical sociopolitical, economic, and geographic contexts. The principle of contextual sensitivity, as argued by Hankivsky, allows for the critical examination of marginalization and oppression through capturing the processes that produce inequities. We heard how nurses who wear a hijab experienced multiple racializing processes in education, practice, and policy. Responsiveness requires a commitment to providing a place for bottom-up theorizing to shift the focus from differences as the problem to “the social constructs that render differences problematic” (Hankivsky, 2004, p. 36). In this way, the nurses’ stories were analyzed in relation to disciplinary power relations. Attentiveness to the consequences of choice offered an understanding of the actions of others that shaped the nurses’ experiences of Islamophobia. We also used ethics of care to contextualize the nurses’ stories within the broader political context of gender, division of labour, and religion. As a political, moral philosophy, care ethics facilitated the contextual understanding of power structures within the discipline with a view towards social justice.

### Data generation

Ethical approval from the Human Research Ethics Board at the University of Victoria (Ethics Protocol Number 20-0185) was obtained before data generation, which was conducted from October 2019 to June 2020. We recruited across Canada to ensure the diversity of participants. The inclusion criteria were: 1) be a registered nurse, licensed practical nurse, or psychiatric registered nurse (RPN); 2) identify as Muslim; 3) wear a hijab in practice; and 4) currently practice in Canada. We employed a variety of purposive sampling and recruitment strategies, including word of mouth, outreach through community faith-based organizations and web-based platforms. The recruitment strategy evolved to address challenges due to difficulty reaching nurses who wear a hijab as a hard-to-reach population and due to the COVID-19 pandemic.

Data were generated through conducting in-depth narrative interviews in person and via telephone. All interviews were conducted by the first author (NS) and averaged between 30 to 90 min. Eight interviews took place during the COVID-19 pandemic between February-June 2020. Only the first interview was conducted in person. This participant did not consent to being recorded however agreed to notes being taken during the interview. The nine remaining interviews were conducted via telephone and audio-recorded. Telephone interviews are conducive to qualitative inquiry and are not necessarily inferior to in-person interviews (Farooq, 2015).

Informed consent was obtained prior to the interviews. At the beginning of each interview, the first author (NS) obtained consent for audio-recording. The voluntary nature of participation was underscored at the beginning, middle, and end of each interview. Each participant was asked: *Can you tell me about your experiences as a Muslim nurse wearing hijab?* This open-ended question was based on the premise that most people like telling stories and with a little encouragement will provide narrative accounts of their experiences (Riessman, 2008). To explore theoretical understandings of racism, an intersectionality lens was applied to dive deeper and to analyze the relationality between racism, class structure, gender and religion (Collins, 2019). Thus, prompts were used such as: *What does it mean for you to wear a hijab? Do you feel like you are singled out in anyway? If so, how?* Ethics of care informed further questions about consequences of action and nurses’ choice and agency, through using prompts such as *what makes you feel like you are supported in practice; Have you experienced any challenges related to wearing your hijab at work?*

### Sample

We interviewed ten nurses (eight registered nurses, one registered nurse who holds an advanced nursing degree and one licensed practical nurse) who work in various Canadian provinces and in different clinical settings providing direct patient care. It is important to note that due to the sensitive nature of the nurses’ stories and the ease with which their identities might be revealed, certain identifying information (i.e., specific geographical locations, educational backgrounds, specific practice sites or specialties) are withheld to protect the identities of the nurses. No compensation was offered to participants.

Determining the sample size for this study was guided by Riessman's (2008) assertion that the exact number of interviews/participants is finalized during data collection and analysis. In qualitative research, the concept of data saturation is often used as determining sample size (Vasileiou et al., 2018). However, we employed Braun and Clarke's (2019) application of saturation in which the concept of ‘information power’ is used to direct a clear relationship between the relevancy of the data and sample size (the more relevant and powerful the data, the fewer participants are needed). Thus, the sample size of this study was determined through the richness and the contextuality of the generated data in meeting the purpose of the study.

### Data analysis

We used Riessman's (2008) case-centered analysis to construct the composite narratives. In case-centered analysis, the story is the case, and when the story is transcribed, it becomes the unit of analysis. Riessman maintains that case-centered approach to data analysis honors the participant's individual agency and consciousness and emphasizes particularities and the contexts; it focuses on attention to details within each narrative, unfolding insights and complexities by paying attention to similarities, differences, and contradictions within and across cases. The first author (NS) read across the transcripts as a whole while being attentive to commonalities, similarities, contradictions, and fragmentation within and across the cases and these were later refined and discussed with the co-authors. We then engaged in “‘compositing’ the process of developing composite characters” to construct a counter-narrative (Patton & Catching, 2009). Compositing is an integral procedure to protect the identities of participants in studies, as this one, where identifying characteristics might unwittingly expose participants who hold particular vulnerabilities in research.

We employed intersectionality as an analytical lens to extend CRF in understanding how social identity factors shaped the nurses’ experiences of anti-Muslim racism. Intersectionality goes beyond the multiplication of social identities (gender, race, class, and religion, to mention a few) to provide an approach to understanding of how certain identities and conditions exist within structures of power (Crenshaw, 1989).

To promote rigour and credibility of the findings, the nurses’ stories are presented in three composite narratives. We purposely constructed each composite narrative to contextualize the experiences of the nurses while weaving together verbatim excerpts from the interviews. We also used theoretical triangulation by using multiple theoretical perspectives to understand the nurses’ experiences. An ‘audit trail’, was used to document the research process and facilitate reflexivity (Lietz & Zayas, 2010).

## Findings

The findings are presented in three composite narratives derived from ten narrative interviews (Table 1). We recognize that there are tensions with grouping the stories of diverse nurses. However, the methodological approach was informed by an intersectional analysis. In this context, strategic essentialism explored diversity and everyday experiences of anti-Muslim racism in nursing. This is akin to strategic essentialism for social justice proposed by Spivak (1988).

**Table 1.** Three composite narratives.

Composite Narratives	Key Storylines
First Composite Narrative: I Am a Muslim Nurse with a Hijab and an Accent	Intersectional identities shaping the experiences of the nurses Having my nursing education and competence questioned Facing covert and overt racism
Second Composite Narrative: Unveiling Operating Power Structures and Power Relations	Equating nurses who wear the hijab with terror and violence Constructing the hijab as unsafe and unclean Being excluded from leadership positions
Third Composite Narrative: Navigating Power Structures and Power Relations	Asserting multiple meanings of the hijab Acting as ambassadors of Islam Reclaiming control

## First composite narrative: I am a muslim nurse with a hijab and an accent

I am a Muslim nurse and I wear the hijab, and I speak with an accent. Unfortunately, throughout my nursing career, I have had harmful experiences because of the way I look and how I speak. I may not feel different, but I am different. I do not see myself as different until it is pointed out; and it is pointed out in subtle and openly hostile ways. When this happens, I stand back and think ‘oh yeah, I’m the nurse with the thing on my head. Frequently, I am reminded that I am different, and that I must remember that I ought to conduct myself differently. Every so often, when I walk into patients’ rooms, when I introduce myself, I am reminded of my differences by the many questions I am asked, which feel more like I am under scrutiny, like hurdles I need to overcome. How long have you worked here? Where did you go to school? Are you forced to wear that? Oh, what is your accent?

This is something that my colleagues and I do not have in common. They are not asked how long they have been nursing and where they got their nursing degrees and what accents they have. No one is asking my colleague from Newfoundland with a very heavy accent where he is from. Usually, when I am asked where I received my nursing education. I cannot shake the undertone of this question which is doubting my competency as a nurse. It has become tiresome to expect these questions and to be on guard, always be prepared to answer as openly as possible. At work in the hospital, I experience subtle racism regularly. While others may not see it that way, it's obvious to me through people's body language and in their mannerisms; like when I began working on a unit and when I would walk into the nurses’ station, the nurses would look the other way. And quite honestly, I’d rather be told directly to ‘take it off’ rather than having a patient, a colleague, or a manager look at me strangely. Along with this subtle racism, I also experience blatant racism. I have been called derogatory racial names and have been singled out by patients to leave the room. I have had my competence and education doubted and questioned. It makes me upset; it makes me feel sorrowful that after all that I do, I am not trusted.

My experiences in nursing school were deeply shaped by there being no other racialized students or educators in my program. It was frustrating when instructors and students would question my ability to comprehend the material or when they seemed to doubt that I was capable of excelling when I received good marks. One instructor wanted to fail me because she thought I could not understand her. That instructor and others have an image of what a good nurse is, a mold that they want their students to fit into, but that I could never resemble or sound like.

## Second composite narrative: unveiling operating power structures and power relations

I was born in Canada, raised in Canada, and educated as a nurse in Canada. But, because I wear the hijab, patients, their family members, and other nurses frequently question where I am from and how long I have lived in Canada. In response, I often explain that I am Canadian, born and raised in Canada, and that I studied in Canada. I am often surprised that I am not always bothered by it; I think maybe it makes me reflect on why this question is being asked. I remember talking to a colleague and apparently, she’d never been asked questions like these. In talking to her, I realized how atypical these types of questions are and how the premise of these questions, to highlight and interrogate my Otherness, is not okay. Of course, I knew that there was something insidious behind these questions, but I didn't grasp the pervasiveness of them in my nursing career against their stark absence from those of my colleagues. Looking back, I can only explain that it is what it is. What was normal to me was absolutely not normal for my nursing colleagues.

Being a Black nurse, I have many experiences of being called racial slurs, including the N-word. The first time, I was shocked and surprised, but chalked it up to the ignorance of the patient. I remember a colleague at the time started crying. She broke down and said, “I’m so sorry that this happened to you”. Other devastating moments were the times I was called a terrorist. The first time it happened was when I went into this patient's room and her son was there. Upon seeing me, seeing my hijab, he refused to have me as his nurse. When I asked him why, his exact answer was that he felt threatened by me because I wear the hijab, that the way I look made him think of all the violence that is happening in the Middle East. Other times, patients will take one look at me and say they do not want me to care for them. “You’re one of them”. Sadly, in the face of these experiences, I rarely feel supported. The next shift after that most recent experience, I was again assigned to this same patient, despite the harm and trauma I had already undergone. I should not have been put back in this unsafe situation. It did him no justice and it did not do me any justice to look after him.

My career goal is to work in a leadership capacity, but I have found no success, though not for a lack of effort. A while ago, I applied for a leadership position and did not even receive an interview, despite me being the only candidate with the required educational background. This was made worse by the lack of transparency on how this decision was made. When I told my colleague about my experience, she said a main consideration among hiring managers is whether a candidate is a good fit with the other leadership members. Well, I do not fit in anywhere. Forget the qualifications, am I the right fit? Will I be someone that they want to chat to about family and pets and their social lives? Well, probably not. We do not lead the same lives.

Another structural challenge I have faced was the implications of the practices within infection prevention and control. An infection control nurse once approached me and questioned my hijab, calling it unclean and unhealthy to patients. I challenged her, asking her, “what is the difference between me washing and changing my hijab before each shift to another nurse who goes home and washes her hair?” Regardless, these concerns have prevented me from working in certain specialties, such as working in the Operating Room (OR). Very recently, during the COVID-19 pandemic, there were concerns about whether I could wear my hijab into the rooms of potentially infected patients or if I had to use an extra hair cover. There were concerns about whether my head scarf invalidated my ability to safely provide care. The rationale was that nurses must cover their hair with a hair cover or a hat and remove it when exiting the room. Therefore, if I were to go into patient rooms, then I would need to take my hijab off upon exiting the room. Alternatively, I could wear a hair bonnet, which is a hair cap over my hijab. But now with the pandemic and the practice of healthcare providers covering their hair and keeping that cover on throughout their shift, it has been kind of nice, because I am sort of an expert as I have been covering my hair for a long time. I am asked by other nurses about what I recommend, how I put my hair up. I feel like it has started conversations and I feel like my colleagues have been thankful for my willingness to answer their questions. It has helped to get rid of the stigma that surrounds Muslim women. Specifically, that our men force us to wear the hijab.

## Third composite narrative: navigating power structures and power relations

I have been a nurse for over a decade. I have practiced in different Canadian provinces. I am also a Muslim woman who wears the hijab. I am clear on the fact that wearing the hijab was my own choice and I often communicate the meanings it holds for me. Wearing the hijab is very much a part of who I am and my identity. I chose to wear it because I believe it is an act of worship and doing so is how I interpret my faith. It is a sign of modesty, and it makes me feel complete and safe and respected in my space. When wearing my hijab, I feel and look beautiful too. As a Muslim woman, I care about how I look, and I can dress it and I can be fashionable. It makes me feel more confident as well, but the main thing is that when I choose to wear a hijab, it is about making that conscious choice to obey Allah's command. Usually, I am the only nurse wearing the hijab on any given unit. I take pride in being the only nurse wearing a hijab in a room because this might be the only experience that someone has with a Muslim woman, and it is nice that it is in the healthcare setting. With this, they can come to learn that Muslim women are everywhere, they are successful, and really in all fields in general. It's nice to be a part of that representation.

I try to take the questions I regularly receive about my hijab and religion as teaching opportunities. To me, it serves as a way of Da’wah in that I get asked questions like ‘why do you wear it?’ and ‘what is it called? I can provide education while presenting Muslim women. It is my belief that through being a good nurse and a good person, I can show the often-untold beauty of Islam. However, it can be challenging to address these questions, even when they are motivated by curiosity. Though I still think religion is a private and personal matter, I am not as put off or shocked when I get asked the questions as I once was. The other interesting and at the same time challenging aspect of my experience is the persistent feeling that, because I am a nurse who wears the hijab and a Black nurse, I always need to work harder than other nurses to prove that I deserve to be a nurse and to assert my normalcy. I often overcompensate by explaining things in full detail to almost prove my knowledge and put those I care for at ease. It says that I know what I’m talking about and I’m here to support you. It's a pervasive need to justify my competence and belonging.

While at work, I am aware that my colleagues can talk politics, current events, and more. I cannot have those conversations because it is not appropriate. I represent an entire group. Recently, a topic came up about the Coronavirus and the Black Lives Matter protests. Some of the nurses I work with called these protests ridiculous and should be stopped as they pose a health threat. What do I say to that? I get the health concerns; I am a nurse working in a hospital during a pandemic; I do not want the ICU flooded with patients. At the same time, I am a Black woman who experiences racism. I understand why the protests are important. It may not be important to them because they do not understand our experiences and what we go through, but I do. My family members do. My friends do. So, I do not discuss politics and I do not talk about religion at work, I do not talk about current events and my views. I sit and listen, and I remind myself that I am not to engage in these conversations, that I choose to be silent.

## Discussion

Across the nurses’ stories, the hijab was a visual representation that rendered its wearers ‘Muslim’. Hampton and Hartman (2019) contend that “whether or not a Muslim woman identifies and/or is racially coded as Black, Brown, White, or otherwise, the hijab itself functions as a racial and racializing signifier” (p. 3). The hijab was fundamental in the construction of the nurses as stranger figures who do not belong; an image that provokes fear and hostility (Zempi & Chakraborti, 2014). It acted as an emblem of their *Otherness*, reinforcing the binary of ‘us’ and ‘them’ and functioned as a “racializing agent” (Selod, 2018, p. 79). This is consistent with studies demonstrating that women who wear a hijab are more likely to face discrimination in the workplace (Aziz, 2012; Koura, 2018; Tariq & Syed, 2017).

The nurses’ experiences conveyed an essentialist portrayal of nurses who wear a hijab as agency-less and oppressed ignores their diversity and their choice. A common master-narrative is that the hijab is forced on women who wear it (Selod, 2018). Questioning the nurses about whether they were coerced to wear the hijab showed how colonial and Orientalist assumptions about the veil as inherently oppressive and a symbol of patriarchal rule have infiltrated the nursing discipline. To the contrary, nurses in our study wear a hijab out of choice, a finding consistent with broader literature on women who veil (Furseth, 2011; Williams & Vashi, 2007). Those who question the autonomy behind the practice of veiling fail to recognize that within a democratic society, such as Canada, it is a fundamental right for women to dress the way they choose. The contextual complexity of the hijab is rooted in historical and gendered hierarchies where, in some Muslim countries, women are pressured to wear it by their families, communities, and the state (Cole & Ahmadia, 2003).

Speaking with a non-native accent emerged as an important axis of difference for many of the nurses who wear a hijab in practice. Matsuda (1991) explains that accents are windows into who we are, the identities we hold, and the lives we live. Accents function as an identity marker, which explains accent-based discrimination. As in all forms of discrimination, power is at the heart of accent discrimination (Guo, 2009; Matsuda, 1991). This form of discrimination can be associated with credentialing and knowledge. Positions of power are maintained through assigning negative stereotypes to those who speak with non-native accents, constructing them as less competent, intelligent, and uneducated (Creese & Kambere, 2003; Pantos & Perkins 2013). Accents also function as a gatekeeper for inclusion or exclusion (Kayaalp, 2016). While the nurses were questioned about their non-native accents, their Canadian colleagues who speak with native accents were not. This delineates who belongs and who does not, which is congruent with the colonial process of constructing the image of the Orient as an outsider and a foreigner (Said, 1978).

Assuming the (in)capability of nurses who wear a hijab by questioning their education and competence is a consequence of equating the hijab with ignorance, wherein women are presumed to have lower levels of education (Rahmath et al., 2016). Similarly, studies on experiences of racialized nurses have illustrated how their education was questioned and their competence was discounted (Allan et al., 2009; Mapedzahama et al., 2012; Whitfield-Harris et al., 2017). The findings of our study suggest that wearing a hijab may be particularly harmful to nurses and pose greater negative consequences. The assumption that nurses who wear a hijab are incapable or under qualified revealed processes of discrimination and exclusion based on the nurses’ Muslim identity, gender and class. Class analysis, as with gender analysis, is central to the experiences of the nurses, as class-based racism is rooted in the history of nursing where “race, gender and class together created nursing as race- and class-stratified ‘women's work’ where the division of work is divided across the racial lines” (Scherzer, 2003, p. 23). Further, the notion of care competence, as articulated by Raghuram (2019), is hierarchical wherein the competencies of some are valued more than those of others. Raghuram argues that race has been central to the division of caring labor in settler colonial states as colonialism has created a racialized division of care, producing and reframing existing racial divisions while using the language of care.

The nurses’ experiences with overt and covert racism were systemic and included their time in the academy. Racism often manifests in subtle forms through attitudes and mannerisms which “can be revealed through heightened suspicion or distrust of a people based on perceived racial difference” (Phillips-Beck et al., 2020, p. 3). Covert racism is harder to call out than that of blatant racism, as it thrives on the uncertainty of the motives behind the treatment of racialized people. In Canadian nursing, it thrives by hiding behind the social liberal values and the professional caring values of the discipline (Hilario et al., 2018). Hence, the nurses’ narratives underscored that the most insidious interactions they had with patients and other nurses with subtle racism, coded in mannerisms, language, and tone of voice. Covert racism can also perpetuate social exclusion (Ferris et al., 2008).

Many of the nurses in this study were perceived by some faculty members, and peers, as incapable of succeeding in their studies. The educational environment within nursing has continued to be unsupportive, unwelcoming and even hostile to racialized students and faculty (Hassouneh, 2006, 2013). Nursing education operate within the academy, the healthcare system, and largely within Canada as a settler colonial power where racial beliefs and practices remain the foundations of these systems (Puzan, 2003). Within these contexts, nursing education and the nursing discipline are stratified along racial lines where White nurses are positioned at the leadership levels and hold the largest bulk of academic assignments (Gustafson, 2007; Van Herk et al., 2011).

The composite narratives capture power structures and power relations in nursing produced by equating the hijab with terror and violence, questioning the nurses about their country of origin, and constructing the hijab as unsafe and unclean with infection control and prevention (ICP) practices. In addition, these processes can perpetuate systemic exclusion of nurses who wear a hijab from leadership positions. The hijab was often associated with violence and terror, leading to equating nurses who wear it with terrorism. This false equivalency is a racializing process embedded in broader social structures that simultaneously construct the hijab as a symbol of oppression and as a threat simultaneously. The racial categorization of the nurses as threatening terrorists illuminated their experiences with Islamophobia, where the Muslim identity is essentialized as connoting a tendency for violence and terrorism (Beydoun, 2016). Importantly, the experiences of the nurses cannot be isolated from the broader legacy of colonization and immigration policies that are premised on credentialing immigrant healthcare providers to strengthen Canada's healthcare system.

Private citizens, including non-racialized nurses, participate in the racialization of Muslims by taking their Canadian citizenship for granted and questioning the nationality of Muslims (Selod, 2018). The stories of the nurses also shine a light on the experiences of Black nurses who veil with blatant and intense racism, including being called the N-word, that is uniquely shaped by the history of anti-Black racism and slavery in Canada. This emphasizes that racialization functions differently based on other intersecting social categories and contexts (Hampton & Hartman, 2019).

Everyday nursing contexts, including ICP practices, revealed both covert and subtle processes of anti-Muslim racism. The hijab within ICP practices has been constructed as a foreign and unclean item of clothing that does not align with what is normalized as a nurse's uniform. Malik and colleagues (2019) explored the experiences of female Muslim healthcare providers wearing a headscarf in the Operation Room (OR) and investigated the mandatory dress code of having ‘arms bare below the elbows’ within the United Kingdom. Their findings delineated multiple challenges for healthcare providers wearing a headscarf in the OR, where some felt embarrassed (23.4%), anxious (37.1%), and bullied (36.5%). These findings have implications on the opportunities available for nurses who wear a hijab within advanced practice as specialized nurses. Hence, at the organizational level, ICP practices continue to operate as a barrier for nurses who wear a hijab from opportunities in some specialized areas.

Notably, despite a gap in research on anti-Muslim racism in nursing, there are emerging voices advocating for the right to veil for healthcare providers, considering that the public was mandated to wear masks that cover a good portion of the face (Ricca, 2020). Syed (2021) argues that the ban on the niqab, for example, has become especially questionable since the beginning of the pandemic wherein masks were a regular, encouraged, and sometimes mandatory practice. The same rationale should be applied to the hijab. As covering one's hair is becoming a required part of Personal Protective Equipment, the tension between the hijab and ICP practices needs to be critically examined, especially within the context of the COVID-19 pandemic where covering one's hair is becoming a part of nurses’ dress to reduce the risk of infection.

The exclusion of Muslim nurses who wear a hijab from leadership positions was a main storyline. Racialized nurses are significantly underrepresented in Canadian nursing, especially at the leadership level and in areas of advanced practice (Premji & Etowa, 2014). Several barriers perpetuate this underrepresentation including racism and tokenism (Gupta, 2008; Vukic et al., 2012). Inherent in this exclusion is the power nursing managers hold not only as people in positions of authority but also as, most often, non-racialized managers who exercise their privilege to exclude racialized bodies. Iheduru-Anderson (2020) explains that maintaining “White comfort”, a culture within nursing in which hiring managers cater to White nurses and attend to their comfort, is a barrier preventing racialized nurses from obtaining leadership positions (p. 667). Further, hiring practices often undermine the appointment of racialized nurses into leadership positions due to what Beard and colleagues (2020) describe as “familiarity bias” in which managers tend to hire nurses “who look, think, and sound like them and appear to share similar worldviews”, and exclude racialized nurses who are ‘not a good fit’ (p. 176). The exclusion of nurses who wear a hijab from leadership holds multiple implications, as it reaffirms the assumptions about them as undereducated and less competent. It also creates a vicious cycle that preserve White spaces within nursing while keeping racialized nurses outside such spaces (Hassouneh et al., 2012; Iheduru-Anderson, 2020).

The nurses leaned into multiple strategies to resist their racialization by articulating the meanings the hijab holds, taking on the role as an ambassador of Islam, controlling the dress, and choosing to silence themselves. The nurses articulated the diverse meanings that assigned to their hijab, which reflected their heterogeneity. Highlighting the meanings of the hijab is also critical in presenting the voices of the nurses who practice within the discipline of Canadian nursing and its prevailing history of systemic racism and discriminatory practice towards racialized nurses (Clark & Saleh, 2019). The meanings were a combination of the hijab as an act of worship, an expression of the Muslim identity, a means of asserting modesty and negotiating public space, and as an expression of beauty and fashion. Our findings echo previous studies that support the hijab as a means of exerting control over their bodies when negotiating public spaces (Litchmore & Safdar, 2016; Siraj, 2011; Zine, 2007). The hijab was also described by the nurses as a source of modesty and beauty, and as a form of fashion. Indeed, the hijab as a representation of modesty does not conflict with the notion of it as a fashionable garment and stands in contrast to its presentation as unfeminine and backward (Michelman, 2003).

To address racializing power relations, nurses took on the role as an ambassador of Islam. They utilized the frequent questions they encountered about their religion and their hijab as a form of ‘*Da’wah*’, an Arabic word that refers to explaining and demonstrating how Islam works for believers. Nurses perceived their experiences as an opportunity to dismantle the master-narratives about them as they contended with wearing a hijab and its social expressions and public representations (Koura, 2018). By embracing their Muslim identity, the nurses demonstrated their agency.

Most of the nurses navigated power relations by controlling their dress and silencing themselves. Controlling the dress takes different forms from removing the hijab to altering it (Selod, 2018). Though none of the nurses in the study removed their hijab, some contemplated removing it to mitigate anti-Muslim racism. The nurses re-signified the hijab by incorporating fashionable trends to contrast with the traditional head cover. Wearing brighter colors, for example, resisted the Western construction of the hijab as a dark garment that symbolizes oppression (Jouili, 2009). Some of the nurses also controlled their dress to mitigate the image of the hijab as unclean and unsafe by opting to wear a smaller hijab and by changing its color daily to visually demonstrate cleanliness. Using Selod's (2018) deconstruction of the complexity of controlling the dress, we argue that in doing so, the nurses reclaimed their agency in asserting control over how their bodies were imagined, pushing against the negative narratives about them. Their stories also revealed the dynamic and interpersonal nature of encounters with institutional structures that perpetuate and reinforce negative portrayal of Muslims (Beydoun, 2016).

The nurses who participated in the study used silence as a refusal to participate in discourses that further racialized them. The Western silence/speech dichotomy views silence as ineffective and speech as positive and empowering (Jungkunz, 2011). However, while Selod (2018) and Lems (2020) connect silencing the self mainly to Muslim men who, out of fear of being associated with terror, remain silent on current events and their political views, Karaman and Christian (2020) studied the experiences of Muslim college women in the US and found that silencing the self was used to mitigate their racialization and protect themselves in highly politicized environments. It is important to understand that the nurses’ choice of silencing themselves was enacted within the context of power imbalances that might render them silent.

## Implications for future research, practice, policy, and education

This study underscores the experiences of nurses who wear a hijab in the context of anti-Muslim racism. The harmful impacts of anti-Muslim racism exemplified by the nurses necessitates research investigating the psychological and physiological effects of Islamophobia. The unique situatedness of the nurses’ required research examining the experiences of Muslim healthcare professionals, including students and gender diverse nurses. Another emerging area of research that requires attention is the credentialing of racialized nurses and the impacts of the COVID-19 pandemic across power hierarchies in specialized and managerial roles in nursing. Moreover, this study brings attention to the contributions of critical theories (Louie-Poon et al., 2022). CRF is emancipatory in that it provides the theoretical space for racialized people to document their stories and to reconstruct narratives. Nursing research grounded in CRF promotes nursing knowledge that privileges diversity of perspectives and is inclusive.

The implications of the findings for nursing practice cannot be separated from implications for policy: institutional change requires formal policies and a commitment at the institutional level. More to the point, care ethics can be used to consider what it means to live together in an antiracist, caring democracy (Lopez & Neely, 2021). Dismantling racism in nursing necessitates a multi-level approach with adequate resources and funding. It is critical that nursing practice and policy are reoriented towards an anti-racist framework that aims to enact transformational change and to advance social justice. Within practice settings, there is an urgent need for creating an open environment for dialogue on racism and its harmful effects where racially based discrimination is expressly understood as unacceptable and reportable.

Finally, there is a need for equitable hiring, promotion, and mentoring policies and practices that use an anti-racist and anti-oppressive lens. Nursing education must also work toward decolonizing approaches that bring together diverse stories to inform the values and structures embedded in nursing curricula, teaching methodologies, and professional development (McGibbon et al., 2014). Thus, settler colonialism and Islamophobia should be openly discussed in nursing education by facilitating dialogue founded on self-reflexivity and which acknowledges individual and structural racism while avoiding the assumption that non-racialized people are racist and paying attention to the context within which racism is perpetuated. The importance of addressing anti-Muslim racism underscores the utility of storytelling as a learning and teaching modality that honors experiential knowledge, gives voice to historically oppressed and silenced individuals and groups, and unsettles the compliance of the dominant group (Bell, 2003). Indeed, nursing education needs to renew its commitment to a care ethics that is directed to achieving social justice.

## Limitations

This study is limited by the lack of representation of RPNs and nurses at leadership levels, mainly due to the underrepresentation of Muslim nurses within managerial roles. Furthermore, expanding the inclusion criteria to include other healthcare providers who wear a hijab, such as physicians, occupational health therapists, and physiotherapists, could have enriched the analysis by providing a comparative context to nursing. However, by restricting the sample to nurses, our aim was to contextualize the experiences of the nurses within the context of the discipline. Finally, the generalizability of the findings is limited as it focuses on a specific group on nurses. Nevertheless, the narratives in this study resonate with broader discourses and documented experiences of racialized nurses, raising the possibility that the findings can be transferred to other groups of nurses.

## Conclusion

This study is a response to the long-standing systemic racism in nursing and adds to understanding anti-Muslim racism in Canadian nursing. We concur with nursing scholars nationally and globally to disrupt racism, which includes anti-Muslim racism. The experiences of the nurses who participated in this study draw attention to the political undertaking of care ethics required to redress anti-Muslim racism through storytelling. We use the nurses’ stories to promote their agency and the transformational change required to address anti-Muslim racism across nursing policies, everyday practice and education.

## Notes

1. We draw on Droogsma's (2007) use of ‘hijab’, ‘veil’, and ‘headscarf’ interchangeably to refer to various forms of head coverings adopted by Muslim women that cover most of a woman's head, ears, arms, and neck. We also acknowledge that though wearing the hijab is an integral aspect of the Muslim identity that is held by Muslim women, its forms continue to evolve and change from one generation to another and between different regions (Bhowon & Bundhoo, 2016).
